# Regioselective Suzuki-Miyaura Reaction: Application to the Microwave-promoted Synthesis of 4,7-Diarylquinazolines

**DOI:** 10.3390/molecules15052949

**Published:** 2010-04-27

**Authors:** Youssef Kabri, Pierre Verhaeghe, Armand Gellis, Patrice Vanelle

**Affiliations:** Laboratoire de Pharmacochimie Radicalaire, Faculté de Pharmacie, Universités d’Aix-Marseille I, II et III - UMR CNRS 6264 , Laboratoire Chimie Provence, 27 Boulevard Jean Moulin, 13385 Marseille cedex 05, France; E-Mails: youssef.kabri@univmed.fr (Y.K.); pierre.verhaeghe@univmed.fr (P.V.); armand.gellis@univmed.fr (A.G.)

**Keywords:** quinazoline, Suzuki-Miyaura reaction, microwaves, S_RN_1

## Abstract

New diarylquinazolines displaying pharmaceutical potential were synthesized in high yields from 4,7-dichloro-2-(2-methylprop-1-enyl)-6-nitroquinazoline by using microwave-promoted regioselective Suzuki-Miyaura cross-coupling reactions.

## Introduction

In 1979, Suzuki and Miyaura introduced organoboron reagents into the realm of cross-coupling chemistry, by demonstrating a palladium-catalyzed reaction of 1-alkenylboranes with aryl and alkynyl halides [1,2]. Since its discovery, this reaction, which is now referred to as the Suzuki-Miyaura reaction, has seen significant advancement and has become one of the most powerful carbon-carbon bond forming methods in organic synthesis [3,4,5,6]. The process has important advantages including functional group compatibility, low toxicity of reagents and intermediates, easy availability of boron derivatives, high thermal stability and good tolerance toward oxygen and aqueous solvents. Recently, organic chemists have turned their work to the application of this reaction to the synthesis of more complex molecules, by using successive Suzuki-Miyaura cross coupling reactions with substrates containing two or more possible reactive sites. One of the solutions to obtain the desired cross-coupling products in a selective manner, by assembling such multi-functionalized compounds, is to favor one of the possible reaction sites [7,8]. Herein, we will describe a regioselective method based on the modulation of the reaction conditions. 

Quinazoline is an important molecular scaffold due to the large variety of pharmacological properties associated with derivatives based on this heterocyclic system [9]. Especially, their importance as selective anticancer chemotherapy agents appears unparalleled and attracts the attention of many pharmaceutical research teams worldwide [10,11,12,13]. Focusing on quinazoline substrates, our group also quite recently described the preparation of new 2-substituted-quinazoline derivatives which exhibit original antiplasmodial properties [14,15,16].

In other respects, microwaves, as a non-conventional source of energy, have become a very popular and useful technology in organic chemistry [17]. The main attraction of using microwaves is the possibility of achieving short reaction times in cleaner systems [18,19,20], even under solventless conditions [21,22]. Microwave irradiation is a simple, rapid and effective method for transferring energy to a polar reaction medium [23]. Consequently, microwave irradiation has been widely applied in organic synthesis, including C-C cross-coupling reactions [24,25].

In continuation of the recent Suzuki-Miyaura monocoupling reactions which we presented in the original 2-(2-methylprop-1-enyl)-6-nitroquinazoline series [26], we investigated the synthesis of a series of new biarylsubstituted-quinazolines, *via* a regioselective Suzuki-Miyaura cross coupling reaction. It was already known in literature [27,28] that the quinazoline C4 position is quite electrophilic. We therefore investigated the possibility of using 4,7-dichloro-2-(2-methylprop-1-enyl)-6-nitroquinazoline (**5**) with various arylboronic acids, aiming at preparing dissymmetric biaryl-substituted quinazolines, taking into account that chlorinated aromatic carbon atoms, at position *α* of a nitro group, also display a high reactivity when involved in palladium-catalyzed coupling reactions [29].

## Results and Discussion

**Scheme 1:**
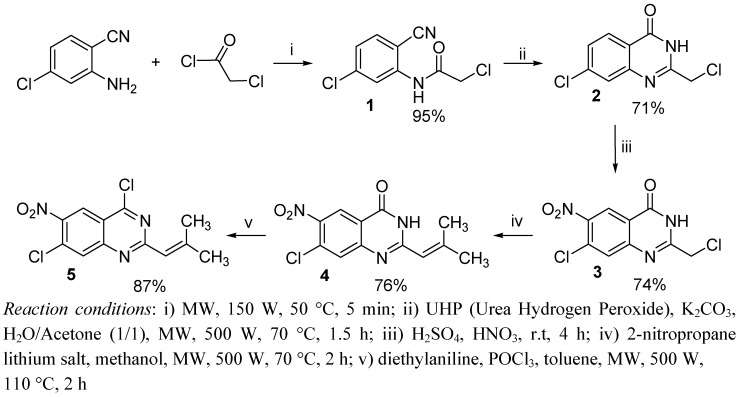
Synthesis of starting material **5**.

The synthesis of the starting material, 4,7-dichloro-2-(2-methylprop-1-enyl)-6-nitroquinazoline (**5**), is presented in Scheme 1. The global synthesis strategy was developed in our research group for the preparation of closely related analogs [30]. Compound **5** was obtained from commercial 2-amino-4-chlorobenzonitrile in good yield after five steps, under microwave irradiation. The first step is the condensation between 2-amino-4-chlorobenzonitrile and chloroacetyl chloride, followed by intramolecular cyclisation, to give product **2** in 67% yield [31]. Then, nitration at the 6 position gave the expected product **3** (74% yield), followed by a S_RN_1 reaction with the lithium salt of 2-nitropropane [32,33] leading to the ethylenic derivative **4** (76% yield). Finally, a microwave-assisted chlorination reaction using phosphorus oxychloride in the presence of *N,N*-diethylaniline gave product **5**, bearing two chlorine atoms in positions 4 and 7 (87% yield).

**Scheme 2:**
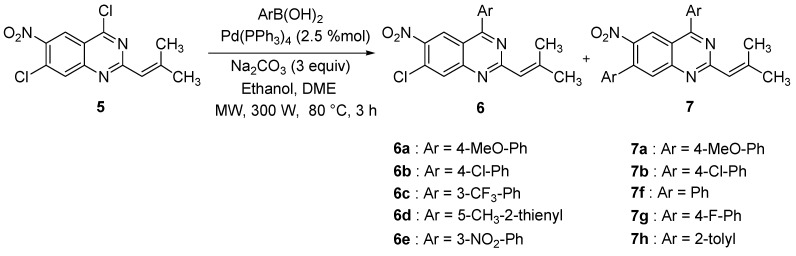
Regioselective Suzuki-Miyaura reaction.

The 4,7-dichloro-2-(2-methylprop-1-enyl)-6-nitroquinazoline (**5**) was engaged in a double Suzuki-Miyaura coupling study with the purpose of identifying reaction conditions which would provide regioselectivity between the 4 and 7 chlorinated positions of the quinazoline ring (Scheme 2). We started by using 4 equiv. of 4-methoxyphenylboronic acid, 4 equiv. of Na_2_CO_3_ and 2.5 mol % of Pd(PPh_3_)_4_. As suggested by Connolly and co-workers [34], a DME/ethanol (9:1, v/v) mixture was used as solvent and the reaction was heated with microwave irradiation.

Under such conditions, only the double-coupled compound **7a** was obtained, in 70% yield, as presented in Table 1 (Entry 1), showing that both positions were reactive as regards of the Suzuki-Miyaura reaction.

To minimize double coupling and substitute at the 4-position selectively, we decreased the amount of arylboronic acid. Using 1.5 equiv. of 4-methoxyphenylboronic acid (Entry 2), some selectivity appeared, the reaction mixture containing both the monosubstituted product **6a** (45% yield) and the disubstituted product **7a** (15% yield). However, a complete selectivity was observed with 1.2 equiv. of 4-methoxyphenylboronic acid. The monosubstituted product **6a** was then obtained in good yield (68%, Entry 3). This result indicates the possibility of achieving a selective coupling reaction at the C4 position, while preventing the chlorinated C7 position from reacting. The monocoupling at the 4-position was checked by NOESY spectrum, where a strong correlation between H-5 (8.65 ppm) and H-2’ (7.77 ppm) was observed.

**Table 1:** Regioselective Suzuki-Miyaura coupling reaction.

Entry	Arylboronic acid Aryl-	Equiv. of Boronic acid	Yield % (6/7)
1	4-MeO-Ph-	4.0	0 / 70
2	4-MeO-Ph-	1.5	45 / 15
3	4-MeO-Ph-	1.2	68 / 0
4	4-Cl-Ph-	4.0	0 / 67
5	4-Cl-Ph-	2.0	42 / 13
6	4-Cl-Ph-	1.5	63 / 0
7	3-CF_3_-Ph-	1.5	48 / 0
8	5-CH_3_-2-thienyl-	2.0	72 / 0
9	5-CH_3_-2-thienyl-	1.5	61 / 0
10	3-NO_2_-Ph-	2.0	55 / 0
11	3-NO_2_-Ph-	1.5	43 / 0

*Reaction conditions*: Pd(PPh_3_)_4_ (2.5 mol %), Na_2_CO_3_ (3-4 equiv), DME/ethanol (9:1), MW 300 W, 80 °C, 3 h

Starting from this initial encouraging result, the influence of both electron-withdrawing and electron-donating substituents on arylboronic acids was investigated next. The reactions with 1.5 equiv. of 4-chlorophenylboronic or 3-trifluoromethylphenylboronic acid provided the monosubstituted products **6b** or **6c** in 63 and 48% yields, respectively (Entries 6 and 7). The reaction of **5** with 3-nitro-phenylboronic acid offered lower reactivity in comparison with the reaction of **5** with 4-methoxy-phenylboronic acid but higher regioselectivity, especially when 2 equiv. of arylboronic acid were used (Entry 10), leading to the monosubstituted product **6e** in 55% yield. A possible explanation for the lower reactivity and good regioselectivity observed when using 3-nitro or 3-trifluorophenylboronic acid and 5-methylthiophen-2-ylboronic acid could result from the chelation of the Lewis basic heteroatoms to the palladium intermediate. Such chelation could be retarding the rate of the reductive elimination step [35]. By using the preceding best conditions for the double coupling reaction, defined for the preparation of compounds **7a** and **7b**, three more symmetric diarylquinazolines **7f-h** were synthesized in good yields (Table 2). 

**Table 2:** Double Suzuki-Miyaura coupling reaction extensions in optimal conditions.

Entry	Aryl-	Product	Yield %
1	Ph-	**7f**	85
2	4-F-Ph-	**7g**	71
3	2-Tolyl-	**7** **h**	65

*Reaction conditions*: arylboronic acid (4 equiv.), Pd(PPh_3_)_4_ (2.5 mol %), Na_2_CO_3_ (4 equiv.), DME/ethanol (9:1), MW 300 W, 80 °C, 3 h

In order to achieve the second coupling reaction at the C7 position of monosubstituted product **6a**, and obtain dissymmetric diarylquinazolines, we started by using 2 equiv. of arylboronic acid, 3 equiv. of Na_2_CO_3_, 2.5 mol % of Pd(PPh_3_)_4_ and a refluxing mixture of DME/ethanol (9:1, v/v). After 3 h, under microwave irradiation, starting material **6a** and the diarylsubstituted product **8** were obtained in a 1:1 ratio (27% yield), indicating the lack of reactivity of the C7 quinazoline position under such reaction conditions, probably due to the insufficient solubility of **6a** in the reaction mixture. 

**Scheme 3:**
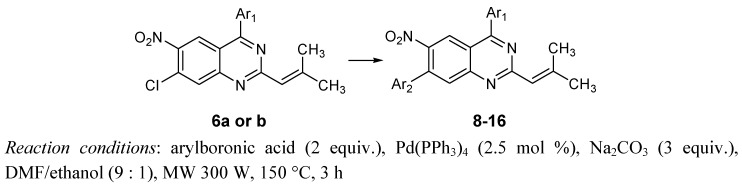
Cross coupling reaction at position 7.

We then proceeded to modify the operating procedure. The DME/ethanol solvent mixture was changed for a DMF/ethanol (9:1) mixture which was refluxed under microwave irradiation (Scheme 3). After 3 h, all the starting material was consumed. A series of dissymmetric diarylquinazolines **8**-**16** was thus synthesized in good yields via this optimized coupling reaction between **6a** or **6b** and various arylboronic acids, as indicated in Table 3.

**Table 3:** Suzuki-Miyaura reactions between monosubstituted products **6a **or **6b** and various arylboronic acids.

Entry	Aryl 1	Aryl 2	Product	Yield %
1	4-MeO-Ph-	4-Cl-Ph-	**8**	55
2	4-MeO-Ph-	Ph-	**9**	64
3	4-MeO-Ph-	4-F-Ph-	**10**	43
4	4-MeO-Ph-	2-Tolyl-	**11**	50
5	4-MeO-Ph-	3-CF_3_-Ph-	**12**	57
6	4-Cl-Ph-	4-MeO-Ph-	**13**	54
7	4-Cl-Ph-	Ph-	**14**	62
8	4-Cl-Ph-	4-F-Ph-	**15**	57
9	4-Cl-Ph-	2-Tolyl-	**16**	58

*Reaction conditions*: arylboronic acid (2 equiv.), Pd(PPh_3_)_4_ (2.5 mol %), Na_2_CO_3_ (3 equiv.), DMF/ethanol (9 : 1), MW 300 W, 150 °C, 3 h

## Experimental

### General

Melting points were determined on a Büchi B-540 apparatus and are uncorrected. Elemental analyses were performed by the Microanalyses Center of the University of Aix-Marseille 3, France. Both ^1^H- and ^13^C-NMR spectra were determined on a Bruker ARX 200 spectrometer. The ^1^H chemical shifts are reported as ppm downfield from tetramethylsilane (Me_4_Si), and the ^13^C chemical shifts were referenced to the solvent peak: CDCl_3_ (76.9 ppm) or DMSO-*d_6_* (39.5 ppm). Solvents were dried by conventional methods. The following adsorbent was used for column chromatography: silica gel 60 (Merck, particle size 0.063-0.200 mm, 70-230 mesh ASTM). TLC was performed on 5 cm × 10 cm aluminium plates coated with silica gel 60F-254 (Merck) in an appropriate solvent.

### Microwave instrumentation

Multimode reactor: ETHOS Synth Lab station and MicroSYNTH Lab terminal 1024 (Ethos start, Milestone Inc.). The multimode microwave has a twin magnetron (2 × 800 W, 2.45 GHz) with a maximum delivered power of 1,000 W in 10 W increments (pulsed irradiation). Built-in magnetic stirring (Teflon-coated stirring bar) was used in all operations. During experiments, time, temperature and power were measured with the “easy WAVE” software package. The temperature was measured throughout the reaction and evaluated by an infrared detector or a optical fiber (ATC-FO 300).

### Synthesis

*2-Chloro-N-(5-chloro-2-cyanophenyl)acetamide* (**1**) [36] and *7-chloro-2-(chloromethyl)quinazolin-4(3H)-one* (**2**) [31] were prepared as previously described. 

### *7-Chloro-2-(chloromethyl)-6-nitroquinazolin-4(3H)-one* (**3**)

To a solution of 7-chloro-2-(chloromethyl)quinazolin-4(3*H*)-one (**2**, 3 g, 13.1 mmol) in concentrated sulfuric acid (40 mL), fuming nitric acid (3.26 mL) was added dropwise at 0 °C. The reaction mixture was stirred at rt for 4 h, poured into crushed ice (100 mL). A precipitate appeared and was filtered, washed with water (3 × 20 mL) and dried in a vacuum drying oven (dessicator cabinet). Recrystallization from propan-2-ol gave 2.66 g. of the title compound. Yield 74%; yellow solid, mp 241 °C. ^1^H-NMR (200 MHz, DMSO-*d_6_*): *δ* = 8.69 (1H, s), 8.07 (1H, s), 4.59 (2H, s); ^13^C-NMR (50 MHz, DMSO-*d_6_*): *δ* = 160.3, 157.0, 151.2, 145.3, 130.9, 130.2, 124.4, 120.7, 43.0; Anal. calcd. for C_9_H_5_Cl_2_N_3_O_3_: C, 39.44; H, 1.84; N, 15.33 %. Found: C, 39.24; H, 1.85; N, 15.26 %.

### *7-Chloro-2-(2-methylprop-1-enyl)-6-nitroquinazolin-4(3H)-one* (**4**)

7-Chloro-2-(chloromethyl)-6-nitroquinazolin-4(3*H*)-one (**3**, 1 g, 3.65 mmol) was added to a solution of the lithium salt of 2-nitropropane (1.37 g, 14.6 mmol) in methanol (40 mL). The reaction mixture was irradiated in a microwave oven, at 70 °C, for 2 h at a power of 500 W. After evaporation of methanol, the residue was dissolved in ethyl acetate (50 mL) and washed with water (3 × 100 mL). The organic layer was dried over magnesium sulphate and the solvent was removed under vacuum. A yellow solid was obtained, which was recrystallized from propan-2-ol to give 7-chloro-2-(2-methylprop-1-enyl)-6-nitro-quinazolin-4(3*H*)-one (**4**) (0.78 g, yield 76%); yellow solid, mp 267 °C. ^1^H‑NMR (200 MHz, DMSO-*d_6_*): *δ* = 8.61 (1H, s), 7.88 (1H, s), 6.06 (1H, s), 2.34 (3H, s), 1.98 (3H, s); ^13^C-NMR (50 MHz, DMSO-*d_6_*): *δ* = 160.6, 155.9, 155.1, 152.2, 143.9, 130.7, 129.8, 124.5, 119.6, 116.9, 28.3, 21.2; Anal. calcd. for C_12_H_10_ClN_3_O_3_: C, 51.53; H, 3.60; N, 15.02 %. Found: C, 51.54; H, 3.64; N, 14.92 %.

### *4,7-Dichloro-2-(2-methylprop-1-enyl)-6-nitroquinazoline* (**5**)

7-Chloro-2-(2-methylprop-1-enyl)-6-nitroquinazolin-4(3*H*)-one (**4**, 0.5 g, 1.79 mmol) was dissolved in toluene (40 mL). Diethylaniline (0.86 mL, 5.37 mmol) and phosphorus oxychloride (0.33 mL, 3.58 mmol) were added. The mixture was heated to 110 °C in the microwave oven irradiating with 500 W for 2 h. After cooling and hydrolysis with 100 mL of water, the mixture was extracted with dichloromethane (3 × 50 mL). The combined organic extracts were washed with water (3 × 100 mL), dried over magnesium sulphate and evaporated. Purification by column chromatography [silica gel, eluent: dichloromethane/petroleum ether (1/2)] afforded 0.46 g of 4,7-dichloro-2-(2-methylprop-1-enyl)-6-nitro-quinazoline (**5**) which recrystallized from propan-2-ol. Yield 87%; brown solid, mp 105 °C. ^1^H-NMR (200 MHz, CDCl_3_): *δ* = 8.69 (1H, s), 8.10 (1H, s), 6.52 (1H, s), 2.42 (3H, s), 2.09 (3H, s); ^13^C-NMR (50 MHz, CDCl_3_): *δ* = 164.3, 162.3, 155.0, 152.2, 146.0, 132.3, 131.3, 123.8, 123.4, 119.1, 28.8, 21.1; Anal. calcd. for C_12_H_9_Cl_2_N_3_O_2_: C, 48.34; H, 3.04; N, 14.09 %. Found: C, 48.42; H, 3.16; N, 13.83 %.

### General procedure of the monocoupling and symmetric double coupling Suzuki-Miyaura reaction

4,7-Dichloro-2-(2-methylprop-1-enyl)-6-nitroquinazoline (**5**, 0.2 g, 0.67 mmol), and tetrakis(tri-phenylphosphine) palladium(0) (2.5 mol %) were dissolved in DME (20 mL) under nitrogen and stirred for 1 h at rt. Arylboronic acid (1.2 to 2 equiv. for monocoupling, 4 equiv. for symmetric double coupling) in ethanol (2 mL) and sodium carbonate (0.21 g, 2.01 mmol: monocoupling, 0.28 g, 2.68 mmol: symmetric double coupling) were added. The mixture was placed in the microwave oven irradiating with 300 W, heating to 80 °C for 3 h. After addition of water (50 mL), the solution was extracted into dichloromethane (3 × 50 mL). The organic layer was washed with water (3 × 100 mL), dried over sodium sulphate and evaporated. The crude product was purified by column chromatography [silica gel, eluent: petroleum ether/ethyl acetate (5%)] and recrystallized from propan-2-ol.

*7-Chloro-4-(4-methoxyphenyl)-2-(2-methylprop-1-enyl)-6-nitroquinazoline* (**6a**). Yield 68%; yellow solid, mp 180 °C. ^1^H-NMR (200 MHz, CDCl_3_): *δ* = 8.65 (1H, s), 8.14 (1H, s), 7.77 (2H, d, *J* = 8.6 Hz), 7.12 (2H, d, *J* = 8.6 Hz), 6.66 (1H, s), 3.93 (3H, s), 2.45 (3H, s), 2.09 (3H, s); ^13^C-NMR (50 MHz, CDCl_3_): *δ* = 168.6, 164.5, 162.1, 152.6, 145.2, 131.8, 131.2, 130.9, 128.3, 125.7, 124.5, 117.8, 114.7, 55.6, 28.6, 21.0; Anal. calcd. for C_19_H_16_ClN_3_O_3_: C, 61.71; H, 4.36; N, 11.36 %. Found: C, 61.49; H, 4.39; N, 11.26 %.

*7-Chloro-4-(4-chlorophenyl)-2-(2-methylprop-1-enyl)-6-nitro quinazoline* (**6b**). Yield 63%; yellow solid, mp 199 °C. ^1^H-NMR (200 MHz, CDCl_3_): *δ* = 8.54 (1H, s), 8.22 (1H, s), 7.74 (2H, d, *J* = 8.5 Hz), 7.60 ( 2H, d, *J* = 8.5 Hz), 6.69 (1H, s), 2.45 (3H, s), 2.11 (3H, s); ^13^C-NMR (50 MHz, CDCl_3_): *δ* = 168.2, 164.4, 153.9, 152.1, 145.5, 137.7, 134.2, 131.4, 131.2, 129.5, 125.0, 124.1, 117.7, 28.8, 21.2; Anal. calcd. for C_18_H_13_Cl_2_N_3_O_2_: C, 57.77; H, 3.50; N, 11.23%. Found: C, 57.54; H, 3.56; N, 11.08 %.

*7-Chloro-2-(2-methylprop-1-enyl)-6-nitro-4-(3-(trifluoromethyl) phenyl)quinazoline* (**6c**)*.* Yield 48%; brown solid, mp 151 °C. ^1^H-NMR (200 MHz, CDCl_3_): *δ* = 8.48 (1H, s), 8.18 (1H, s), 8.07 (1H, s), 7.95-7.88 ( 2H, m), 7.79-7.72 (1H, m), 6.66 (1H, s), 2.45 (3H, s), 2.11 (3H, s); ^13^C-NMR (50 MHz, CDCl_3_): *δ* = 167.6, 164.7, 153.2, 152.7, 145.6, 136.8, 132.9, 131.9, 131.8, 131.3, 129.6, 127.5, 126.7, 124.6, 124.4, 123.6, 117.7, 28.6, 21.0; Anal. calcd. for C_19_H_13_ClF_3_N_3_O_2_: C, 55.96; H, 3.21; N, 10.30 %. Found: C, 55.85; H, 3.22; N, 10.14 %.

*7-Chloro-2-(2-methylprop-1-enyl)-4-(5-methylthiophen-2-yl)-6-nitro quinazoline* (**6d**)*.* Yield 72%; yellow solid, mp 172 °C. ^1^H-NMR (200 MHz, CDCl_3_): *δ* = 8.99 (1H, s), 8.08 (1H, s), 7.67 (1H, d, *J* = 3.9 Hz), 6.97 (1H, d, *J* = 3.9 Hz), 6.57 (1H, s), 2.62 (3H, s), 2.45 (3H, s), 2.09 (3H, s); ^13^C NMR (50 MHz, CDCl_3_): *δ* = 164.3, 160.9, 152.9, 152.8, 148.2, 145.2, 137.9, 132.8, 131.3, 131.0, 127.7, 124.9, 124.2, 116.5, 28.7, 21.1, 15.8; Anal. calcd. for C_17_H_14_ClN_3_O_2_S: C, 56.74; H, 3.92; N, 11.68 %. Found: C, 56.64; H, 4.01; N, 11.48 %.

*7-Chloro-2-(2-methylprop-1-enyl)-6-nitro-4-(3-nitrophenyl)quinazoline* (**6e**)*.* Yield 55%; brown solid, mp 197 °C. ^1^H-NMR (200 MHz, CDCl_3_): *δ* = 8.68 (1H, s), 8.52-8.47 (2H, m), 8.24 (1H, s), 8.09 (1H, d, *J* = 7.6 Hz), 7.87-7.79 (1H, m), 6.70 (1H, s), 2.46 (3H, s), 2.12 (3H, s); ^13^C-NMR (50 MHz, CDCl_3_): *δ* = 166.7, 164.6, 154.1, 152.5, 148.8, 145.7, 137.6, 135.4, 131.7, 131.6, 130.2, 125.5, 124.8, 124.2, 124.1, 117.5, 28.8, 21.2; Anal. calcd. for C_18_H_13_ClN_4_O_4_: C, 56.19; H, 3.41; N, 14.56 %. Found: C, 56.16; H, 3.47; N, 14.35 %.

*4,7-Bis(4-methoxyphenyl)-2-(2-methylprop-1-enyl)-6-nitroquinazoline* (**7a**). Yield 70%; yellow solid, mp 175 °C. ^1^H-NMR (200 MHz, CDCl_3_): *δ* = 8.58 (1H, s), 8.07 (1H, s), 7.84 (2H, d, *J* = 8.8 Hz), 7.37 (2H, d, *J* = 8.9 Hz), 7.14 (2H, d, *J* = 8.8 Hz), 7.00 (2H, d, *J* = 8.9 Hz), 6.72 (s, 1H), 3.94 (3H, s), 3.87 (3H, s), 2.46 (3H, s), 2.10 (3H, s); ^13^C-NMR (50 MHz, CDCl_3_): *δ* = 168.4, 164.0, 161.9, 160.2, 152.4, 147.5, 140.1, 131.8, 131.2, 129.2, 128.8, 128.7, 124.8, 124.1, 118.2, 116.1, 114.6, 114.5, 55.6, 55.4, 28.5, 20.9; Anal. calcd. for C_26_H_23_N_3_O_4_: C, 70.73; H, 5.25; N, 9.52 %. Found: C, 70.23; H, 5.35; N, 9.48 %.

*4,7-Bis(4-chlorophenyl)-2-(2-methylprop-1-enyl)-6-nitroquinazoline* (**7b**). Yield 67%; yellow solid, mp 233 °C. ^1^H-NMR (200 MHz, CDCl_3_): *δ* = 8.56 (1H, s), 8.08 (1H, s), 7.79 (2H, d, *J* = 8.4 Hz), 7.62 (2H, d, *J* = 8.4 Hz), 7.47 (d, 2H, d, *J* = 8.5 Hz), 7.35 (2H, d, *J* = 8.5 Hz), 6.72 (1H, s), 2.46 (3H, s), 2.11 (3H, s); ^13^C-NMR (50 MHz, CDCl_3_): *δ* = 168.4, 163.7, 153.7, 151.5, 147.2, 140.0, 137.7, 135.4, 134.7, 134.4, 131.3, 129.5, 129.3, 129.2, 124.0, 123.9, 118.3, 28.8, 21.2; Anal. calcd. for C_24_H_17_Cl_2_N_3_O_2_: C, 64.01; H, 3.81; N, 9.33 %. Found: C, 63.57; H, 3.80; N, 9.17 %. 

*2-(2-Methylprop-1-enyl)-6-nitro-4,7-diphenylquinazoline* (**7f**)*.* Yield 85%; yellow solid, mp 176 °C. ^1^H-NMR (200 MHz, CDCl_3_): *δ* = 8.58 (1H, s), 8.08 (1H, s), 7.86-7.81 (2H, m), 7.65-7.61 (3H, m), 7.51-7.41 (5H, m), 6.71 (1H, s), 2.46 (3H, s), 2.10 (3H, s); ^13^C-NMR (50 MHz, CDCl_3_): *δ* = 169.2, 164.1, 152.2, 151.7, 147.4, 140.7, 136.5, 136.3, 131.7, 130.8, 130.0, 129.1, 128.9, 127.8, 124.7, 124.1, 118.5, 28.5, 20.9; Anal. calcd. for C_24_H_19_N_3_O_2_: C, 75.57; H, 5.02; N, 11.02 %. Found: C, 75.36; H, 5.02; N, 10.95 %. 

*4,7-Bis(4-fluorophenyl)-2-(2-methylprop-1-enyl)-6-nitroquinazoline* (**7g**). Yield 71%; yellow solid, mp 193 °C. ^1^H-NMR (200 MHz, CDCl_3_): *δ* = 8.55 (1H, s), 8.06 (1H, s), 7.89-7.82 (2H, m), 7.44-7.29 (4H, m), 7.22-7.14 (2H, m), 6.70 (1H, s), 2.46 (3H, s), 2.11 (3H, s); ^13^C-NMR (50 MHz, CDCl_3_): *δ* = 168.0, 164.4, 164.1, 163.2, 152.2, 152.1, 147.2, 139.8, 132.4, 132.3, 132.2, 132.0, 131.8, 129.8, 129.6, 124.5, 123.9, 118.3, 116.6, 116.3, 116.1, 115.9, 28.6, 20.9; Anal. calcd. for C_24_H_17_F_2_N_3_O_2_: C, 69.06; H, 4.11; N, 10.07 %. Found: C, 68.89; H, 4.33; N 9.90 %. 

*2-(2-Methylprop-1-enyl)-6-nitro-4,7-bis(2-tolyl)quinazoline* (**7h**)*.* Yield 65%; yellow solid, mp 161 °C. ^1^H-NMR (200 MHz, CDCl_3_): *δ* = 8.25 (1H, s), 7.97 (1H, s), 7.53-7.35 (5H, m), 7.33-7.28 (2H, m), 7.24-7.16 (1H, m), 6.70-7.72 (1H, m), 2.44 (3H, m), 2.26 (3H, s), 2.18 (3H, s), 2.08 (3H, m); ^13^C-NMR (50 MHz, CDCl_3_): *δ* = 170.9, 164.2, 152.1, 151.7, 147.3, 141.1, 136.5, 136.2, 135.5, 135.4, 132.1, 131.2, 130.2, 130.0, 129.6, 128.7, 128.3, 126.1, 125.9, 124.7, 123.9, 119.7, 28.6, 20.9, 20.1, 20.0; Anal. calcd. for C_26_H_23_N_3_O_2_: C, 76.26; H, 5.66; N, 10.26 %. Found: C, 75.88; H, 5.74; N, 10.12 %.

### General procedure of dissymmetric coupling Suzuki-Miyaura reaction of the 7-position

7-Chloro-4-arylquinazoline (**6**, 0.54 mmol), and tetrakis(triphenylphosphine) palladium(0) (2.5 mol %) were dissolved in DMF (30 mL) under nitrogen and stirred for 1 h at rt. Arylboronic acid (2 equiv., 1.08 mmol) in ethanol (2 mL) and sodium carbonate (3 equiv., 1.62 mmol) were added. The mixture was placed in the microwave oven irradiating with 300 W, heating to 150 °C for 3 h. After addition of water (50 mL), the solution was extracted into dichloromethane (3 × 50 mL). The organic layer was washed with water (3 × 100 mL), dried over sodium sulphate and evaporated. The crude product was purified by column chromatography [silica gel, eluent: petroleum ether/ethyl acetate (10%)] and recrystallized from propan-2-ol. 

*7-(4-Chlorophenyl)-4-(4-methoxyphenyl)-2-(2-methylprop-1-enyl)-6-nitroquinazoline* (**8**). Yield 55%; yellow solid, mp 186 °C. ^1^H-NMR (200 MHz, CDCl_3_): *δ* = 8.66 (1H, s), 8.01 (1H, s), 7.83 (2H, d, *J* = 8.7 Hz), 7.46 (2H, d, *J* = 8.5 Hz), 7.36 (2H, d, *J* = 8.5 Hz), 7.14 (2H, d, *J* = 8.7 Hz), 6.69 (1H, s), 3.94 (3H, s), 2.46 (3H, s), 2.10 (3H, s); ^13^C-NMR (50 MHz, CDCl_3_): *δ* = 168.6, 164.1, 162.1, 152.4, 151.9, 146.8, 139.4, 135.1, 131.9, 131.6, 129.2, 128.6, 124.7, 118.5, 114.7, 55.6, 28.6, 21.0; Anal. calcd. for C_25_H_20_ClN_3_O_3_: C, 67.34; H, 4.52; N, 9.42 %. Found: C, 67.54; H, 4.67; N, 9.34 %.

*4-(4-Methoxyphenyl)-2-(2-methylprop-1-enyl)-6-nitro-7-phenyl quinazoline* (**9**)*.* Yield 64%; yellow solid, mp 165 °C. ^1^H-NMR (200 MHz, CDCl_3_): *δ* = 8.63 (1H, s), 8.02 (1H, s), 7.83 (2H, d, *J* = 8.9 Hz), 7.49-7.41 (5H, m), 7.13 (2H, d, *J* = 8.9 Hz), 6.67 (1H, s), 3.93 (3H, s), 2.45 (3H, s), 2.09 (3H, s); ^13^C- NMR (50 MHz, CDCl_3_): *δ* = 168.4, 164.1, 162.0, 152.5, 151.1, 147.2, 140.5, 136.7, 131.8, 131.7, 128.9, 128.8, 128.7, 127.8, 124.9, 124.3, 118.4, 114.6, 55.6, 28.5, 20.9; Anal. calcd. for C_25_H_21_N_3_O_3_: C, 72.98; H, 5.14; N, 10.21 %. Found: C, 72.70; H, 5.17; N, 10.06 %.

*7-(4-Fluorophenyl)-4-(4-methoxyphenyl)-2-(2-methylprop-1-enyl)-6-nitroquinazoline* (**10**)*.* Yield 43%; yellow solid, mp 180 °C. ^1^H-NMR (200 MHz, CDCl_3_): *δ* = 8.64 (1H, s), 8.03 (1H, s), 7.84 (2H, d, *J* = 8.7 Hz), 7.44-7.37 (2H, m), 7.22-7.12 (4H, m), 6.70 (1H, s), 3.94 (3H, s), 2.46 (3H, s), 2.10 (3H, s); ^13^C-NMR (50 MHz, CDCl_3_): *δ* = 168.9, 163.6, 163.2, 162.2, 151.5, 147.2, 139.8, 132.4, 131.9, 130.9, 129.8, 129.6, 128.5, 124.5, 124.0, 118.3, 116.3, 115.8, 114.7, 55.6, 28.7, 21.1; Anal. calcd. for C_25_H_20_FN_3_O_3_: C 69.92; H, 4.69; N, 9.78 %. Found: C, 68.63; H, 4.89; N, 9.74 %. HRMS-FAB: m/z [M + H]^+^ calcd. for C_25_H_20_FN_3_O_3_: 429.1489; Found: 429.1561.

*4-(4-Methoxyphenyl)-2-(2-methylprop-1-enyl)-6-nitro-7-o-tolyl quinazoline* (**11**)*.* Yield 50%; yellow solid, mp 191 °C. ^1^H-NMR (200 MHz, CDCl_3_): *δ* = 8.60 (1H, s), 8.02 (1H, s), 7.83 (2H, d, *J* = 8.8 Hz), 7.35-7.28 (4H, s), 7.13 (2H, d, *J* = 8.8 Hz), 6.67 (1H, s), 3.93 (3H, s), 2.45 (3H, s), 2.43 (3H, s), 2.09 (3H, s); ^13^C-NMR (50 MHz, CDCl_3_): *δ* = 168.3, 164.1, 161.9, 152.6, 150.8, 147.4, 140.5, 138.9, 133.7, 131.8, 131.6, 129.7, 128.9, 127.7, 124.9, 124.2, 118.3, 114.6, 55.6, 28.5, 21.3, 20.8; Anal. calcd. for C_26_H_23_N_3_O_3_: C, 73.39; H, 5.45; N, 9.88 %. Found: C, 72.87; H, 5.64; N, 9.65 %.

*4-(4-Methoxyphenyl)-2-(2-methylprop-1-enyl)-6-nitro-7-(3-(trifluoro methyl)phenyl)quinazoline* (**12**). Yield 57%; yellow solid, mp 116 °C. ^1^H-NMR (200 MHz, CDCl_3_): *δ* = 8.73 (1H, s), 7.99 (1H, s), 7.84 (2H, d, *J* = 8.8 Hz), 7.73-7.67 (2H, m), 7.64-7.55 (2H, m), 7.13 (2H, d, *J* = 8.8 Hz), 6.66-6.67 (1H, m), 3.93 (3H, s), 2.46 (3H, m), 2.08 (3H, m); ^13^C-NMR (50 MHz, CDCl_3_): *δ* = 168.5, 164.4, 162.0, 152.7, 151.6, 146.3, 139.0, 137.7, 132.1, 131.8, 131.3, 131.1, 129.2, 128.6, 125.4, 124.9, 124.8, 124.7, 123.8, 118.6, 114.6, 55.5, 28.5, 20.9; Anal. calcd. for C_26_H_20_F_3_N_3_O_3_: C, 65.13; H, 4.20; N, 8.76 %. Found: C, 65.00; H, 4.12; N 8.52 %.

*4-(4-Chlorophenyl)-7-(4-methoxyphenyl)-2-(2-methylprop-1-enyl)-6-nitroquinazoline* (**13**). Yield 54%; yellow solid, mp 173 °C. ^1^H-NMR (200 MHz, CDCl_3_): *δ* = 8.46 (1H, s), 8.07 (1H, s), 7.78 (2H, d, *J* = 8.4 Hz), 7.61 (2H, d, *J* = 8.4 Hz), 7.37 (2H, d, *J* = 8.6 Hz), 7.01 (2H, d, *J* = 8.6 Hz), 6.69 (1H, s), 3.87 (3H, s), 2.45 (3H, s), 2.10 (3H, s); ^13^C-NMR (50 MHz, CDCl_3_): *δ* = 169.0, 163.4, 162.7, 160.6, 152.2, 148.1, 141.4, 137.9, 134.4, 131.4, 129.6, 129.5, 129.3, 129.1, 127.9, 123.5, 117.9, 114.6, 55.4, 29.0, 21.4; Anal. calcd. for C_25_H_20_ClN_3_O_3_: C, 67.34; H, 4.52; N, 9.42 %. Found: C, 66.90; H, 4.58; N, 9.19 %.

*4-(4-Chlorophenyl)-2-(2-methylprop-1-enyl)-6-nitro-7-phenyl quinazoline* (**14**). Yield 62%; yellow solid, mp 204 °C. ^1^H-NMR (200 MHz, CDCl_3_): *δ* = 8.53 (1H, s), 8.10 (1H, s), 7.79 (2H, d, *J* = 8.4 Hz), 7.61 (2H, d, *J* = 8.4 Hz), 7.49-7.41 (5H, m), 6.71 (1H, s), 2.45 (3H, s), 2.11 (3H, s); ^13^C-NMR (50 MHz, CDCl_3_): *δ* = 167.9, 164.1, 152.4, 151.9, 147.5, 140.9, 137.3, 136.4, 134.8, 131.9, 131.3, 129.4, 128.9, 127.8, 124.6, 123.6, 118.2, 28.6, 20.9; Anal. calcd. for C_24_H_18_ClN_3_O_2_: C, 69.31; H, 4.36; N, 10.10 %. Found: C, 68.91; H, 4.46; N, 9.94 %.

*4-(4-Chlorophenyl)-7-(4-fluorophenyl)-2-(2-methylprop-1-enyl)-6-nitro quinazoline* (**15**)*.* Yield 57%; yellow solid, mp 211 °C. ^1^H-NMR (200 MHz, CDCl_3_): *δ* = 8.54 (1H, s), 8.11 (1H, s), 7.79 (2H, d, *J* = 8.5 Hz), 7.62 (2H, d, *J* = 8.5 Hz), 7.44-7.37 (2H, m), 7.23-7.14 (2H, m), 6.74 (1H, s), 2.46 (3H, s), 2.12 (3H, s); ^13^C-NMR (50 MHz, CDCl_3_): *δ* = 168.1, 164.1, 163.2, 152.6, 152.1, 147.3, 139.9, 137.5, 134.6, 132.4, 131.7, 131.3, 129.7, 129.6, 129.5, 124.4, 123.8, 118.3, 116.3, 115.9, 28.7, 21.1; Anal. calcd. for C_24_H_17_ClFN_3_O_2_: C, 66.44; H, 3.95; N, 9.69 %. Found: C, 66.28; H, 4.11; N, 9.53 %.

*4-(4-Chlorophenyl)-2-(2-methylprop-1-enyl)-6-nitro-7-o-tolyl quinazoline* (**16**). Yield 58%; yellow solid, mp 192 °C. ^1^H-NMR (200 MHz, CDCl_3_): *δ* = 8.49 (1H, s), 8.08 (1H, s), 7.79 (2H, d, *J* = 8.4 Hz), 7.61 (2H, d, *J* = 8.4 Hz), 7.35-7.28 (4H, m), 6.70 (1H, s), 2.45 (3H, s), 2.43 (3H, s), 2.10 (3H, s); ^13^C- NMR (50 MHz, CDCl_3_): *δ* = 167.7, 164.1, 152.4, 151.5, 147.6, 140.8, 139.0, 137.2, 134.8, 133.4, 131.7, 131.2, 129.7, 129.4, 127.6, 124.7, 123.4, 118.1, 28.5, 21.3, 20.9; Anal. calcd. for C_25_H_20_ClN_3_O_2_: C, 69.85; H, 4.69; N, 9.77 %. Found: C, 69.21; H, 4.81; N, 9.52 %.

## Conclusions

We have reported herein an efficient and regioselective access to symmetric and dissymmetric 4,7-diarylquinazolines by using the Suzuki-Miyaura reaction on the 4,7-dichloroquinazoline derivative **5**, under microwave irradiation. The regioselectivity was controlled by modifying both the amount of arylboronic acid used and the nature of the reaction medium. This method opens the way to a general synthesis of *bis*-functionalized quinazolines, skeletons of great interest for designing biologically active compounds.
